# Supplementary material for: Disentangling choice value and choice conflict in sequential decisions under risk
Source: PLoS Comput Biol. 2022 Oct 7;18(10):e1010478. doi: 10.1371/journal.pcbi.1010478 (PMC9581387; doi:10.1371/journal.pcbi.1010478)
Supplement: S1 Text — Fig A: Top panel: Percentage of decisions before and after the IP for a simulated group of 100 players with different risk preferences (risk averse on average, with α = .8). The decisions before the IP significantly decrease with the probability of losing, making the task more balanced. Lower panel: Percentage of decisions after the IP as a function of the IP, for the same group of simulated participants as in C. Participants with higher IPs (more risk seeking) experience a more imbalanced task in the 2/6 and 1/6 conditions. Fig B: Joint parameter distribution used for the simulation of 100 participants risk averse on average (mean α = .8). These parameters were used for the simulation reported in S1 Text. Fig C: Joint parameter distribution used for the simulation of 100 participants risk neutral on average (mean α = 1). These parameters were used for the simulation reported in the main text. (PDF) [file pcbi.1010478.s001.pdf]

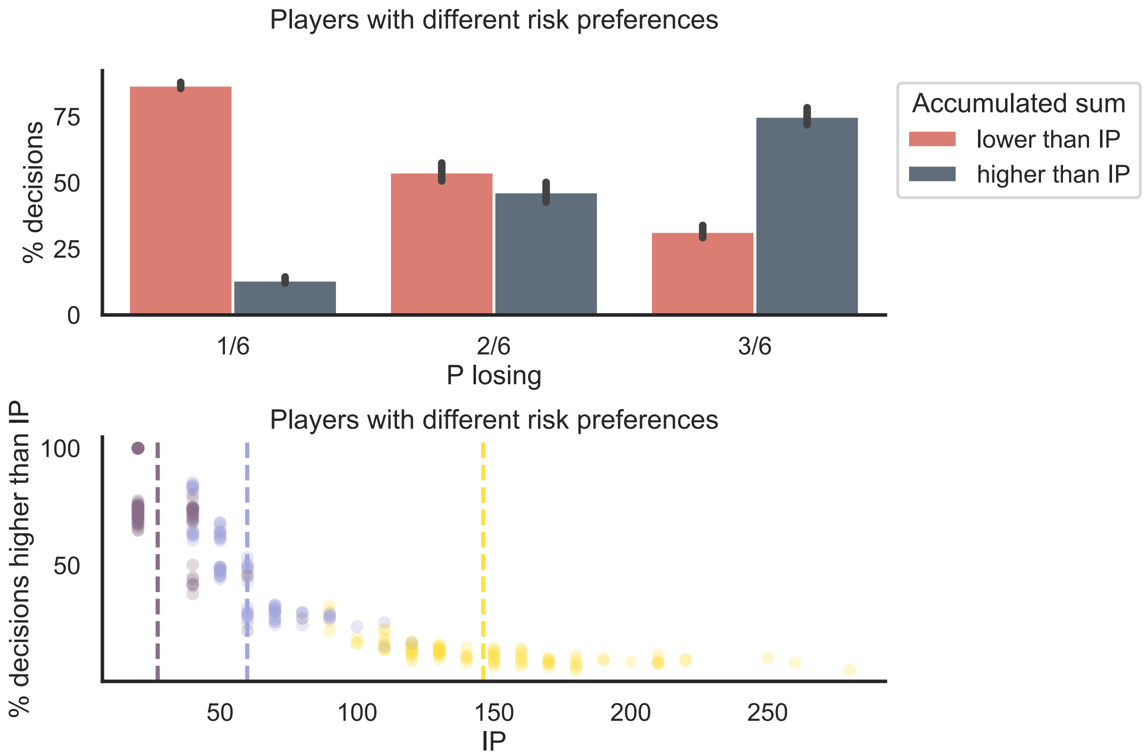

Figure A. **Top panel**: Percentage of decisions before and after the IP for a simulated group of 100 players with different risk preferences (risk averse on average, with α=.8). The decisions before the IP significantly decrease with the probability of losing, making the task more balanced. **Lower panel**: Percentage of decisions after the IP as a function of the IP, for the same group of simulated participants as in C. Participants with higher IPs (more risk seeking) experience a more imbalanced task in the 2/6 and 1/6 conditions.

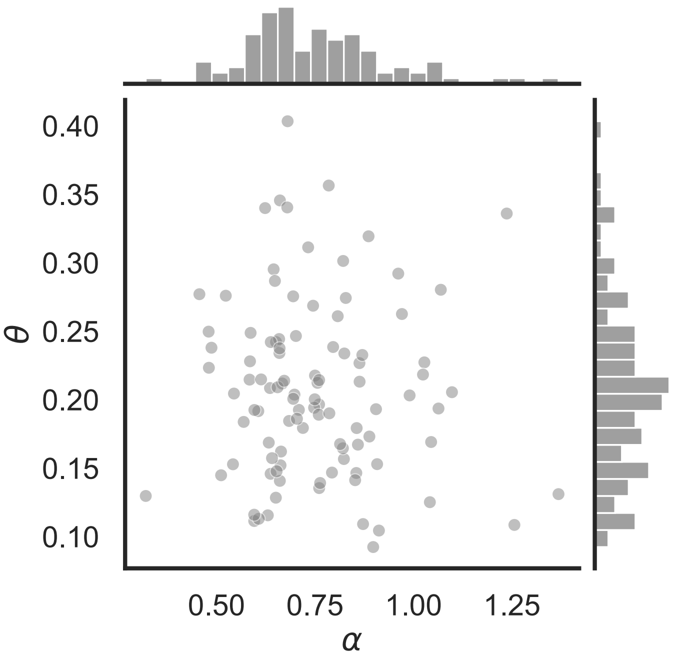

Figure B
Joint parameter distribution used for the simulation of 100 participants risk averse on average (mean $\alpha = .8$). These parameters were used for the simulation reported in the Figure A in S1 Text.

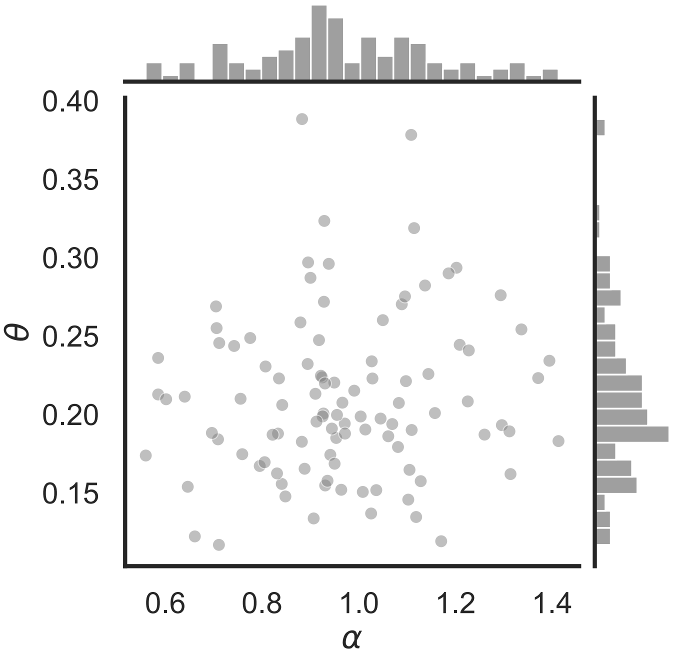

Figure C

Joint parameter distribution used for the simulation of 100 participants risk neutral on average (mean α=1). These parameters were used for the simulation reported in the Results section.
