# Supplementary material for: Disentangling choice value and choice conflict in sequential decisions under risk
Source: PLoS Comput Biol. 2022 Oct 7;18(10):e1010478. doi: 10.1371/journal.pcbi.1010478 (PMC9581387; doi:10.1371/journal.pcbi.1010478)
Supplement: S2 Text — Table A: Logistic regression coefficients summary. Note. Summary of the intercept and cumulative sum coefficients at the group level. Table B: Linear regression coefficients summary. Note. Summary of the intercept, cumulative sum of rewards, decision number, and round number coefficients at the group level. Table C: Diffusion decision model coefficients summary. Note. The IP was calculated based on the drift-rate coefficients alone (IP =−δ0/δ1). The μ HDI are reported for all the diffusion decision model coefficients, that describe the effect of the cumulative sum on the main parameters: the drift-rate δ, the threshold A, and the relative starting point z. The model was only fit to the data of Experiment 2. (PDF) [file pcbi.1010478.s002.pdf]

Table A

Logistic regression coefficients summary.

Experiment	Condition	Coefficient	μ HDI	σ HDI
1	1/6	intercept	[-5.7 -3.87]	[1.52 2.95]
1	1/6	cumulative sum	[0.03 0.05]	[0.02 0.03]
2	1/6	intercept	[-6.9 -5.4]	[1.68 2.99]
2	1/6	cumulative sum	[0.03 0.05]	[0.02 0.04]
2	2/6	intercept	[-6.97 -5.01]	[2.27 3.88]
2	2/6	cumulative sum	[0.06 0.09]	[0.03 0.06]
2	3/6	intercept	[-4.89 -2.92]	[2.26 3.91]
2	3/6	cumulative sum	[0.09 0.14]	[0.05 0.1]

Note. Summary of the intercept and cumulative sum coefficients at the group level.

Table B

Linear regression coefficients summary.

Experiment	Condition	Coefficient	μ HDI	σ HDI
1	1/6	intercept	[-0.34 -0.21]	[0.14 0.24]
1	1/6	cumulative sum	[-0.004 0.049]	[0.035 0.075]
1	1/6	decision number	[0.02 0.07]	[0.02 0.06]
1	1/6	round number	[-0.070 -0.001]	[0.067 0.117]
2	1/6	intercept	[-0.52 -0.27]	[0.24 0.48]
2	1/6	cumulative sum	[0.03 0.07]	[0.04 0.08]
2	1/6	decision number	[0.02 0.06]	[0.03 0.07]
2	1/6	round number	[-0.0021 -0.0006]	[0.002 0.003]
2	2/6	intercept	[-0.38 -0.16]	[0.26 0.46]
2	2/6	cumulative sum	[-0.02 0.06]	[0.07 0.14]
2	2/6	decision number	[0.02 0.08]	[0.03 0.1]
2	2/6	round number	[-0.001 0.0001]	[0.001 0.002]
2	3/6	intercept	[-0.35 -0.17]	[0.19 0.35]
2	3/6	cumulative sum	[-0.16 -0.05]	[0.10 0.19]
2	3/6	decision number	[-0.02 0.10]	[0.11 0.22]
2	3/6	round number	[-0.0007 0.0004]	[0.001 0.002]

Note. Summary of the intercept, cumulative sum of rewards, decision number, and round number coefficients at the group level.

Table C

Diffusion decision model coefficients summary.

Condition:	1/6	2/6	3/6
IP(low)	115	61.67	27.5
IP(high)	190.98	89.19	45.91
δ_1(low)	0.02	0.04	0.05
δ_1(high)	0.03	0.05	0.07
δ_0(low)	-4.11	-3.91	-2.73
δ_0(high)	-3.33	-3.03	-1.74
A_1(low)	-0.001	-0.0018	0.0008
A_1(high)	0.0002	0.001	0.0061
A_0(low)	1.33	1.36	1.23
A_0(high)	1.57	1.61	1.47
z_1(low)	-0.002	-0.006	-0.009
z_1(high)	-0.001	-0.003	-0.005
z_0(low)	0.04	0.18	0.19
z_0(high)	0.22	0.35	0.37

Note. The IP was calculated based on the drift-rate coefficients alone (IP = - δ_0/δ_1). The μ HDI are reported for all the diffusion decision model coefficients, that describe the effect of the cumulative sum on the main parameters: the drift-rate δ, the threshold A, and the relative starting point z. The model was only fit to the data of Experiment 2.
