# Supplementary material for: Disentangling choice value and choice conflict in sequential decisions under risk
Source: PLoS Comput Biol. 2022 Oct 7;18(10):e1010478. doi: 10.1371/journal.pcbi.1010478 (PMC9581387; doi:10.1371/journal.pcbi.1010478)
Supplement: S3 Text — Fig A: Distribution of R squared based on 4000 posterior samples for the logistic and linear regressions in Experiment 1. Fig B: Distribution of R squared based on 4000 posterior samples for the logistic and linear regressions in Experiment 2. (PDF) [file pcbi.1010478.s003.pdf]

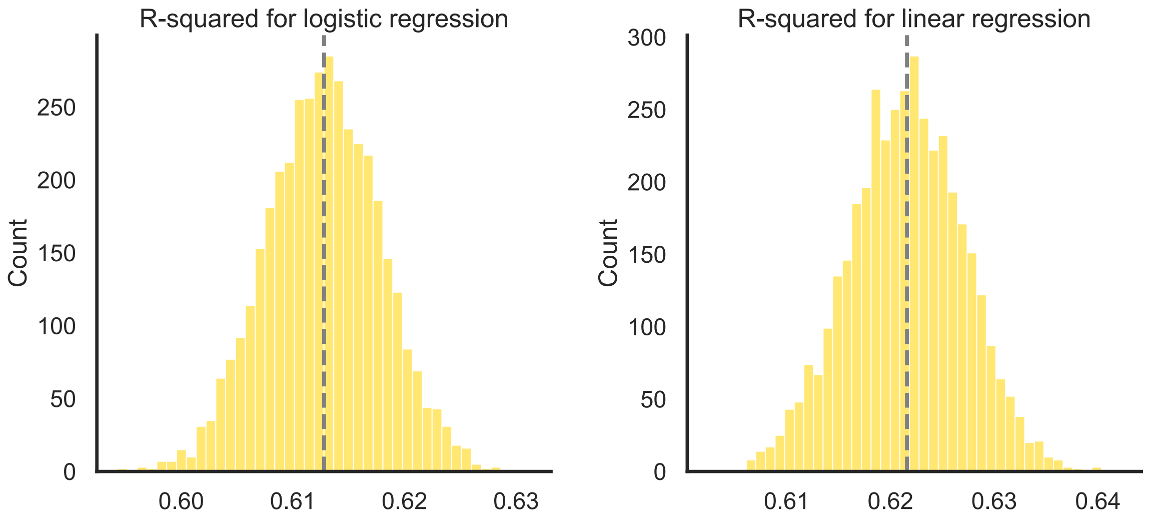

Figure A. Distribution of R squared based on 4000 posterior samples for the logistic and linear regressions in Experiment 1.

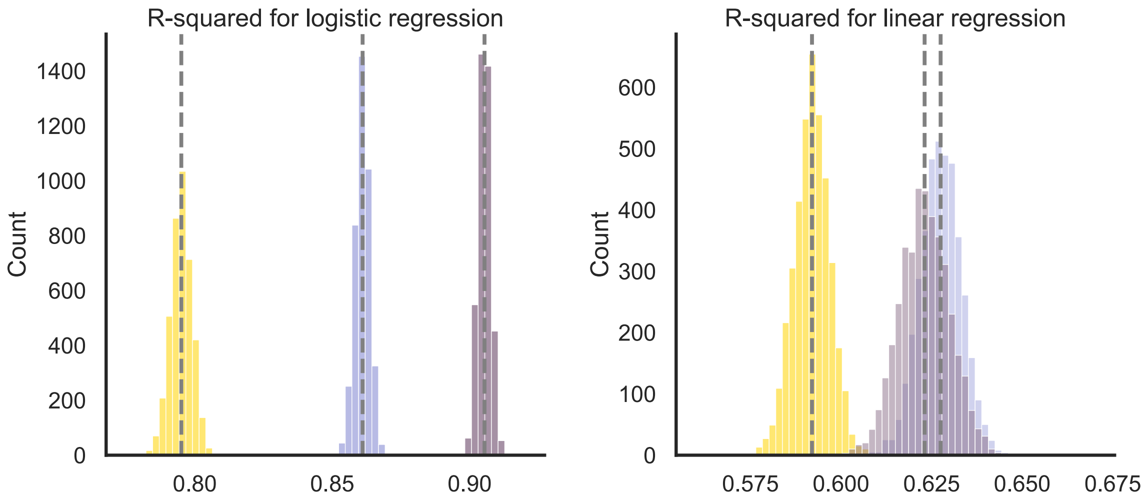

Figure B. Distribution of R squared based on 4000 posterior samples for the logistic and linear regressions in Experiment 2.
