# Supplementary material for: Disentangling choice value and choice conflict in sequential decisions under risk
Source: PLoS Comput Biol. 2022 Oct 7;18(10):e1010478. doi: 10.1371/journal.pcbi.1010478 (PMC9581387; doi:10.1371/journal.pcbi.1010478)
Supplement: S4 Text — Additional regression models Additional analyses to check whether a logistic regression model in which the condition was treated as a continuous predictor, together with the cumulative sum of rewards and their interaction, could explain the data better. (PDF) [file pcbi.1010478.s004.pdf]

Additional regression models

In the main analyses, we fitted the logistic and linear regression model separately across conditions, meaning that we estimated separated intercept and slopes for the three versions of the dice game that the participants played (p=1/6, p=2/6, p=3/6). However, we ran additional analyses to check whether a logistic regression model in which the condition was treated as a continuous predictor, together with the cumulative sum of rewards and their interaction, could explain the data better.

The logistic regression model reported in our main analyses had a lower WAIC (WAIC=10515, -lppd=5038, p_{WAIC}=219, WAIC$_{se}$=142) than the regression model with the condition as a continuous predictor (WAIC=10852, -lppd=5267, p_{WAIC}=159, WAIC$_{se}$=144).

The estimated parameters of the logistic regression model with conditions as a continuous predictor all differed from 0: the intercept had 95% Bayesian Credible Intervals (BCI) equal to [-9.2; -6.6], the cumulative sum of rewards coefficient had 95% BCI=[0.003 0.031], the probability of losing (i.e., condition) coefficient had 95% BCI=[0.9 1.8] and the inter-action coefficient had 95% BCI=[0.02 0.04]. Therefore, the higher the cumulative sum of rewards, the higher the probability to decide to stop; The probability of stopping increased also with the probability of losing (i.e., the condition, coded as 1,2, and 3, respectively for the conditions p=1/6, p=2/6, and p=3/6), and there was a positive interaction between the cumulative sum of rewards and the probability of losing, indicating that participants were more likely to stop after collecting some rewards in the conditions with higher loss probability.

While the interpretation of the two regression models does not differ much, we prefer the model that has separate regression coefficients per condition, as it imposes less constraints on the relationship between the different game conditions and also fits the data better, according to the WAIC.
