# Supplementary material for: Disentangling choice value and choice conflict in sequential decisions under risk
Source: PLoS Comput Biol. 2022 Oct 7;18(10):e1010478. doi: 10.1371/journal.pcbi.1010478 (PMC9581387; doi:10.1371/journal.pcbi.1010478)
Supplement: S5 Text — Table A: Model comparison of diffusion decision models (DDM) based on WAIC (data: Experiment 2). Note. If a DDM parameter was not modulated by any variable, a single value was estimated for it. If it was modulated, an intercept and a coefficient variable were estimated per variable. Lower WAICs indicate better fits to data after accounting for model complexity. **Best model. *These models do not fit credibly worse than the best model (because of the relatively high WAICse). (PDF) [file pcbi.1010478.s005.pdf]

Additional diffusion decision models

Here we report the full model comparison, including the models in which the drift-rate is modulated by the decision number (i.e., models 11 to 19) and a base model in which none of the parameters are modulated (i.e., model 1). Overall, the best fitting models are the models presented in Figure 6, in which the drift rate is modulated by the cumulative sum of rewards, and among these the best fitting ones are the ones in which the starting point is also modulated by the cumulative sum of rewards.

Table A

Model comparison of diffusion decision models (DDM) based on WAIC (data: Experiment 2).

ID	Drift-rate	Starting-point	Threshold	p_{WAIC}	-lppd	WAIC	WAIC_{se}
1	not modulated	not modulated	not modulated	486	6552	14076	273
2	cumulative sum	not modulated	not modulated	615	2903	7036	286
3	cumulative sum	not modulated	decision number	643	2821	6929	287
4	cumulative sum	not modulated	cumulative sum	652	2775	6854	288
5	cumulative sum	decision number	not modulated	646	2831	6955	286
6	cumulative sum	decision number	decision number	662	2776	6877	287
7	cumulative sum	decision number	cumulative sum	678	2713	6782*	288
8	cumulative sum	cumulative sum	not modulated	652	2705	6714*	290
9	cumulative sum	cumulative sum	decision number	683	2625	6616*	291
10	cumulative sum	cumulative sum	cumulative sum	688	2601	6577**	292
11	decision number	not modulated	not modulated	602	3979	9162	288
12	decision number	not modulated	decision number	630	3902	9065	289
13	decision number	not modulated	cumulative sum	625	3910	9071	288
14	decision number	decision number	not modulated	623	3917	9080	290
15	decision number	decision number	decision number	648	3855	9004	290
16	decision number	decision number	cumulative sum	642	3858	9001	290
17	decision number	cumulative sum	not modulated	635	3848	8965	286
18	decision number	cumulative sum	decision number	654	3780	8870	287
19	decision number	cumulative sum	cumulative sum	647	3804	8903	287

Note. If a DDM parameter was not modulated by any variable, a single value was estimated for it. If it was modulated, an intercept and a coefficient variable were estimated per variable. Lower WAICs indicate better fits to data after accounting for model complexity. **Best model. *These models do not fit credibly worse than the best model (because of the relatively high WAIC_{se}).
