# Supplementary material for: Disentangling choice value and choice conflict in sequential decisions under risk
Source: PLoS Comput Biol. 2022 Oct 7;18(10):e1010478. doi: 10.1371/journal.pcbi.1010478 (PMC9581387; doi:10.1371/journal.pcbi.1010478)
Supplement: S6 Text — Fig A: Posterior distribution of the group-level IP according to the logistic model and to the best fitting diffusion decision model in the second Experiment, separately for the different conditions. Note that the IP in the diffusion model is calculated based on the drift rate coefficients only. (PDF) [file pcbi.1010478.s006.pdf]

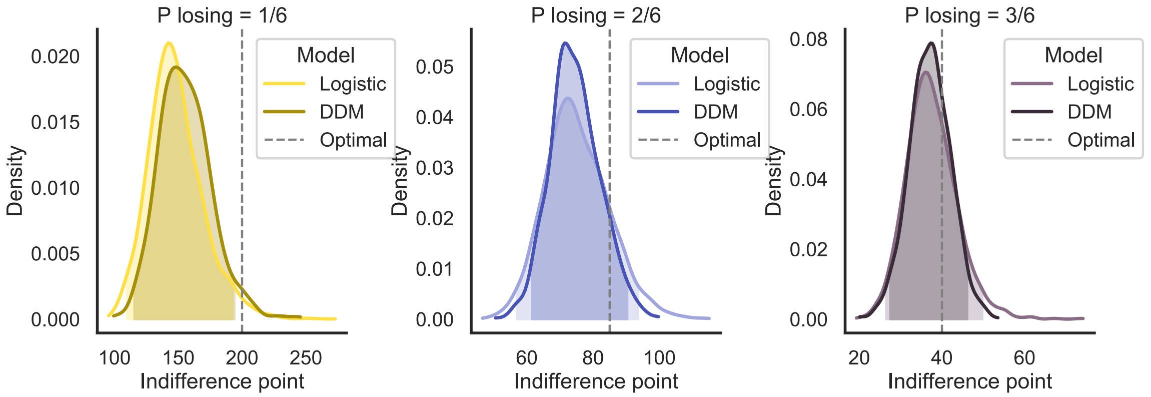

Figure A . Posterior distribution of the group-level IP according to the logistic model and to the best fitting diffusion decision model in the second Experiment, separately for the different conditions. Note that the IP in the diffusion model is calculated based on the drift rate coefficients only.
